# Editorial: Women in Oral Infections and Microbes: 2021

**DOI:** 10.3389/froh.2022.958630

**Published:** 2022-06-15

**Authors:** Diane M. Daubert

**Affiliations:** Department of Periodontics, School of Dentistry, University of Washington, Seattle, WA, United States

**Keywords:** oral health, eriodictyl, maternal inflammation, oral microbiome, immune cell populations

It is our pleasure to see the topic “*Women in Oral Infections and Microbes*” receiving focus in Frontiers. There are many talented women who conduct research in the field of oral health, and this is an opportunity to highlight some specific achievements. This Research Topic includes a series of four articles. One study investigates the effect of eriodictyl on the pathogenicity of *Porphyromonas gingivalis*. The second provides a review of the role of maternal infections and subsequent response on the craniofacial development of the fetus. The third characterizes the oral microbiome in a rural vs. urban Indonesian population, and the final article evaluates the oral immune environment in non-human primates to present data on site specific immunongenic or tolergenic responses. The wide variety of novel research highlights the work of women in oral health research.

Eriodictyl is a natural molecule synthesized by plants that has been used in traditional medicinal practice. It has shown some promise as a natural alternative for the management of periodontal disease. Maquera-Huacho et al. investigated the effect of *Porphyromonas gingivalis* induced reactive oxygen species (ROS) from gingival keratinocytes when in the presence of eriodictyl. Their aim was to determine the dose-dependent effect of eriodictyl on the production of ROS and to assess cytokine and matrix metalloproteinase production by macrophages. They found that eriodictyl attenuated the production of ROS and dose-dependently inhibited the production of MMPs. Their findings support the potential use of eriodictyl as a therapeutic agent for preventing or treating periodontal disease.

Bhagirath et al. provide an extensive review article on the effects of maternal infection and subsequent inflammatory response on the craniofacial development of the fetus. They review the immunological activation and regulation during pregnancy since the mother must not mount an immune response to the developing fetus which displays paternal major histocompatibility complex antigens, yet at the same time the mother must activate an immune response to maternal infections. They discuss regulators of inflammatory responses in pregnancy on fetal development and explore literature on pathogens as possible epigenetic modifiers. Lastly, they review the effect of maternal infections on fetal neurogenesis and craniofacial defects.

The influence of geographical and socio-economic factors on the oral microbiome was investigated by Widyarman et al. in a pilot study of women from Indonesia. Twenty women were included in the study and were evaluated. Tongue samples were collected from 10 rural and 10 urban women and high-throughput DNA sequencing was performed. A significant difference was found in the bacterial community profiles between the two groups. They conclude that the differences may be linked to specific dietary, cultural, and socio-economic differences between the two groups.

The characterization of human immune cells in the oral environment in specific niches has not been thoroughly characterized in homeostasis. Hernandez et al. utilized non-human primates (NHP) as a model for the human immune system and oral anatomy. They collected cytobrush and biopsy samples from buccal, sublingual, and lingual tonsil and made comparisons by sex and age. Tonsil tissues had significantly more T-cells and B-cells than buccal tissues. They found that tonsil tissues were statistically similar across sex and age differences. The characterization may help provided information for the development of oral mucosal immunotherapies in the future.

In conclusion, this Research Topic includes original and review papers showcasing the work of women in oral health research. Women have often been underrepresented as authors and this issue provides a window into the rich variety of work by women.

## Author Contributions

DD wrote the manuscript and approved the final version of the manuscript.

## Conflict of Interest

The author declares that the research was conducted in the absence of any commercial or financial relationships that could be construed as a potential conflict of interest.

## Publisher's Note

All claims expressed in this article are solely those of the authors and do not necessarily represent those of their affiliated organizations, or those of the publisher, the editors and the reviewers. Any product that may be evaluated in this article, or claim that may be made by its manufacturer, is not guaranteed or endorsed by the publisher.

